# Healthcare access and barriers to utilization among transgender and gender diverse people in Africa: a systematic review

**DOI:** 10.1186/s44263-024-00073-2

**Published:** 2024-06-27

**Authors:** Abbas Jessani, Teagan Berry-Moreau, Reeya Parmar, Alexia Athanasakos, Jessica L. Prodger, Andrew Mujugira

**Affiliations:** 1 Department of Dentistry, Schulich School of Medicine and Dentistry, Western University, London, ON Canada; 2 Department of Epidemiology and Biostatistics, Schulich School of Medicine and Dentistry, Western University, London, ON Canada; 3 Department of Microbiology and Immunology, Schulich School of Medicine and Dentistry, Western University, London, ON Canada; 4 Department of Global Health, University of Washington, Seattle, WA USA; 5 Infectious Diseases Institute, Makerere University, Kampala, Uganda

**Keywords:** Transgender and gender diverse people, Africa, Healthcare access, Gender-affirming care

## Abstract

**Background:**

Transgender and gender diverse (TGD) people face significant challenges in accessing timely, culturally competent, and adequate healthcare due to structural and systemic barriers, yet there is a lack of research exploring the access and utilization of healthcare services within African TGD communities. To address this gap, this systematic review explored: (1) barriers to accessing healthcare services and gender-affirming hormone therapy (GAHT) faced by TGD people, (2) demographic and societal factors correlated with the utilization of healthcare services and GAHT, (3) common healthcare and support services utilized by TGD people, and (4) patterns of accessing healthcare services and GAHT within TGD communities.

**Methods:**

A systematic literature search was conducted in PubMed, Embase, and Scopus in September 2023. Eligible studies included peer-reviewed original research, reports, and summaries published in the English language assessing health service accessibility and utilization of TGD people in Africa between January 2016 and December 2023.

**Results:**

From 2072 potentially relevant articles, 159 were assessed for eligibility following duplicate removal, and 49 were included for analysis. Forty-five articles addressed barriers to accessing healthcare services and GAHT, seven focused on demographic and societal factors correlated with the utilization of healthcare services and GAHT, 16 covered common healthcare and support services utilized by TGD people, and seven examined patterns of accessing healthcare services and GAHT. Findings suggested a limited availability of health services, inadequate knowledge of TGD healthcare needs among healthcare providers, a lack of recognition of TGD people in healthcare settings, healthcare-related stigma, and financial constraints within African TGD communities. An absence of studies conducted in Northern and Central Africa was identified.

**Conclusions:**

TGD people in Africa encounter significant barriers when seeking healthcare services, leading to disparity in the utilization of healthcare and resulting in a disproportionate burden of health risks. The implications of these barriers highlight the urgent need for more high-quality evidence to promote health equity for African TGD people.

**Trial registration:**

PROSPERO CRD42024532405.

**Supplementary Information:**

The online version contains supplementary material available at 10.1186/s44263-024-00073-2.

## Background

Transgender and gender diverse (TGD) people face numerous structural and systemic barriers that hinder their ability to obtain timely, culturally competent, and appropriate healthcare services. Consequently, they are disproportionately affected by disparities in social, physical, and mental health when compared to their cisgender counterparts [1]. Previous research has found that negative experiences within healthcare settings and the fear of discrimination often cause TGD people to delay, avoid, or completely forgo seeking essential healthcare services [2–4]. This leads to higher levels of unmet healthcare treatment needs among TGD people, including mental health disorders, systemic problems such as healthcare worker ignorance, biases in clinic structure, forms, and electronic medical record systems, as well as adverse oral health conditions, such as a greater prevalence of dental decay and periodontal diseases [5, 6]. While these barriers exist, to some extent, for TGD people globally, the specific challenges faced by this population in Africa are of particular concern [7].

A lack of research on TGD populations in Africa is largely witnessed, and at present, there is limited data available on the prevalence of TGD people across the continent [8]. Southern Africa is one of the five African regions to report that 0.3% of its total population self-identifies as TGD, underscoring a significant gap in TGD-focused literature throughout Northern, Eastern, Western, and Central Africa [9]. It is suggested that the lack of TGD people in research from Africa is related to the criminalization of same-sex behaviour and minority genders in many African countries and the subsequent fear of adverse repercussions from participation in research [8].

For centuries, precolonial African societies did not view gender as a binary, nor did they correlate anatomy to gender identity [10]. For example, during precolonial times, the *mudoko dako*, effeminate males among the Langi of northern Uganda, were treated as women and could marry men [10]. The *Mwami* prophets of the Ila people in Zambia were men who dressed as women and formed intimate connections with women without engaging in sexual intercourse [10]. Among the Lugbara people of Northwestern Uganda, TGD people often carried out communication with the spirit world, with TGD women and men named *okule* and *agule*, respectively [10]. However, colonization and the spread of fundamentalist Christian attitudes by the British meant that much of Africa lost its previous cultural and spiritual attitudes towards dualistic gender identity [11]. Transgressive gender performances became the target of efforts to “civilize” African societies, leading to the creation of crimes that once did not exist, and the forcing of conformity to Westernized idealized depictions of femininity and masculinity [10, 11].

Now, generations later, gender is socially constructed and regulated, even within healthcare systems, thereby manifesting as an ideological framework that reinforces the negative perceptions of gender nonconformity or incongruence between sex and gender [12]. Within African healthcare systems shaped by this gender structure—biases and norms confine TGD people within an institutionalized gender binary, disregarding the fluidity and diversity of gender identities and expressions [12]. Such attitudes are upheld in Western healthcare systems as well, with individuals being classified into two socially and biologically distinct categories: male-assigned persons who are expected to identify as boys and men and perform masculinity, and female-assigned persons who are expected to identify as girls and women and perform femininity [13]. It was not until recently that many Western healthcare systems came to acknowledge gender diversity as integral to individual dignity and common humanity; however, despite growing support for the protection of gender identity and sexual orientation, there has been slow recognition of the needs of TGD people [13]. This consequently fosters healthcare provider (HCP) ignorance, hindering TGD people from accessing essential care, advice, or proper referrals for transition and gender-affirming care (GAC) [12, 14]. This stigmatization appears rooted in power dynamics and broader societal discourses within public health systems that perpetuate the discursive portrayal of TGD people as pathological and socially deviant [12, 14].

Adding to the complexity of this situation is the enactment of discriminatory lesbian, gay, bisexual, transgender, queer, and other gender identities (LGBTQ +) policies in African countries such as Uganda, Ghana, Namibia, Niger, and Tanzania, which have garnered global notoriety for their severity [4, 15]. Anti-homosexuality laws penalize those identifying as LGBTQ + , contributing to a cycle of stigma, homonegativity, and discrimination [10]. As a result, TGD people who perceive their behaviours to be associated with shame, judgment, fear, or even legal consequences are less likely to access and utilize healthcare services, increasing their risk of adverse health outcomes [15].

GAC, ranging from gender-affirming surgeries and gender-affirming hormone therapy (GAHT) to mental health support, is fundamental in improving the overall health, psychological well-being, and self-fulfillment of many TGD people [16]. GHAT can either masculinize or feminize the body and is a common component of GAC for TGD people [16]. Although GAHT would ideally be administered within a supportive healthcare system managed by a primary HCP, this is absent in most sub-Saharan African countries, including, but not limited to, Uganda, Kenya, and South Africa [17]. This discrepancy raises significant concerns regarding the potential adverse effects and heightened health risks due to improper use or lack of access to GAHT, highlighting a critical gap in healthcare services for TGD people within these countries [18, 19].

Amidst the widely recognized disparities faced by TGD people in healthcare, research exploring access to and utilization of healthcare services within African TGD communities remains limited. This systematic review seeks to explore this gap in the literature by consolidating evidence and exploring: (1) barriers to accessing healthcare services and GAHT faced by TGD people, (2) demographic and societal factors correlated with the utilization of healthcare services and GAHT, (3) common healthcare and support services utilized by TGD people, and (4) patterns of accessing healthcare services and GAHT within TGD communities.

## Methods

### Search strategy and study selection

The protocol for this systematic review was retrospectively registered with the International Prospective Register of Systematic Reviews, PROSPERO (ID: CRD42024532405). The Preferred Reporting Items for Systematic Reviews and Meta-Analyses (PRISMA) checklist was utilized as a guideline for the workflow of this systematic review; details can be found in Additional file 1: Table S1.

In September 2023, an electronic search was conducted using three databases (PubMed, Embase, and Scopus) to identify relevant articles. Forward and backward citation tracing was conducted on the included articles to ensure no potentially pertinent articles were overlooked in the database search. Search terms comprised relevant keywords and controlled vocabulary. The search strategy for all three databases is provided in Additional file 1: Table S2. The titles and abstracts of the articles produced from the database searches, as well as grey literature sources (from non-traditional publishing channels, including reports, policy papers, government documents, etc.), were imported into COVIDENCE. A grey literature search strategy was developed to incorporate the results from customized Google searches. This involved manual searches utilizing the search terms provided in Additional file 1: Table S2 to ensure that a wide range of relevant documents, reports, and non-traditional sources were identified. These sources included various government and organizational websites, policy documents, dissertations, literature reviews, and other relevant resources [20]. The approach aimed to complement the findings from the scientific and peer-reviewed databases, providing a more comprehensive view of the available evidence [20]. The COVIDENCE tool streamlines evidence synthesis steps of the systematic review process, including citation importing, screening, quality assessment, and data extraction [15, 21]. Following de-duplication, two reviewers independently screened the titles and abstracts for potential inclusion and then independently screened the remaining full-text records against specified eligibility criteria, resolving any disparities during screening through discussion and consensus. Fifty studies from COVIDENCE were accepted into this systematic review.

### Inclusion and exclusion criteria

The eligibility criteria were determined in advance and were relevant in the screening and selection of articles. Studies were included if they were peer-reviewed original research, reports, perspectives, or summaries assessing health service accessibility and utilization of African populations self-identifying as TGD. The studies must have also been published in the English language between January 2016 and December 2023. These inclusion criteria were selected to decrease ambiguity and ensure no study was excluded without a thorough screening. Studies were excluded if they only assessed healthcare access during the COVID-19 pandemic, broadly assessed LGBTQ + populations without specifying TGD data, assessed structural interventions designed to eliminate barriers to care and assessed disease incidence and prevalence.

### Data extraction and risk of bias assessment

Data describing the characteristics of each study, including the author, year of publication, country, study design, type of study, type of sampling, main objectives, and results, were extracted and then verified by two reviewers (TBM and RP) using a standard form on Microsoft Excel (Version 16.70). Risk of bias assessment utilized the CLARITY tool for cross-sectional and cohort studies, Joanna Briggs Institute (JBI) for qualitative studies, systematic reviews, scoping reviews, and the Authority, Accuracy, Coverage, Objectivity, Date and Significance** (**AACODS) checklist for evaluating and critically appraising grey literature [22–24]. Qualitative studies, excluding cross-sectional ones, underwent evaluation using the JBI Critical Appraisal Checklist. Articles utilizing a qualitative, quantitative, or mixed methods cross-sectional design were assessed using the CLARITY cross-sectional tool. The JBI Critical Appraisal Checklist was used to evaluate and address potential biases in all studies that met the inclusion criteria [23]. Grey literature was evaluated using the AACODS checklist, a tool designed to critically assess the quality of the information found in grey literature based on criteria such as authority, accuracy, coverage, objectivity, date, and significance [25]. The risk of bias in each study was assessed by utilizing five domains of the CLARITY tool: (1) representativeness of the source population, (2) adequacy of response rate, (3) proportion of missing data, (4) comprehensiveness, clarity, and face validity of the survey, and (5) reliability and construct validity of the survey.

Figure S1 presents a detailed risk of bias assessment for each study. A domain was marked as “no information” if the study did not explicitly provide related information. In cross-sectional studies, the categories “probably no” and “probably yes” were combined and labeled as “moderate” risk of bias. The integration of grey literature and secondary literature was considered in the context of the review’s objectives, acknowledging potential biases and inherent limitations in these sources. One qualitative study was excluded from this review based on its risk of bias score, resulting in 49 studies accepted into this systematic review.

### Data synthesis

Since the main purpose of this review was to explore the access and utilization of healthcare services within African TGD communities, data was synthesized through narrative discussion. According to the objectives of this review and due to the wide variety of reported beliefs in the included studies, a meta-analysis could not be conducted. The included studies were also descriptive in nature, providing only information pertaining to the barriers, utilization, and/or access to healthcare services among African TGD people.

## Results

**Fig. 1:**
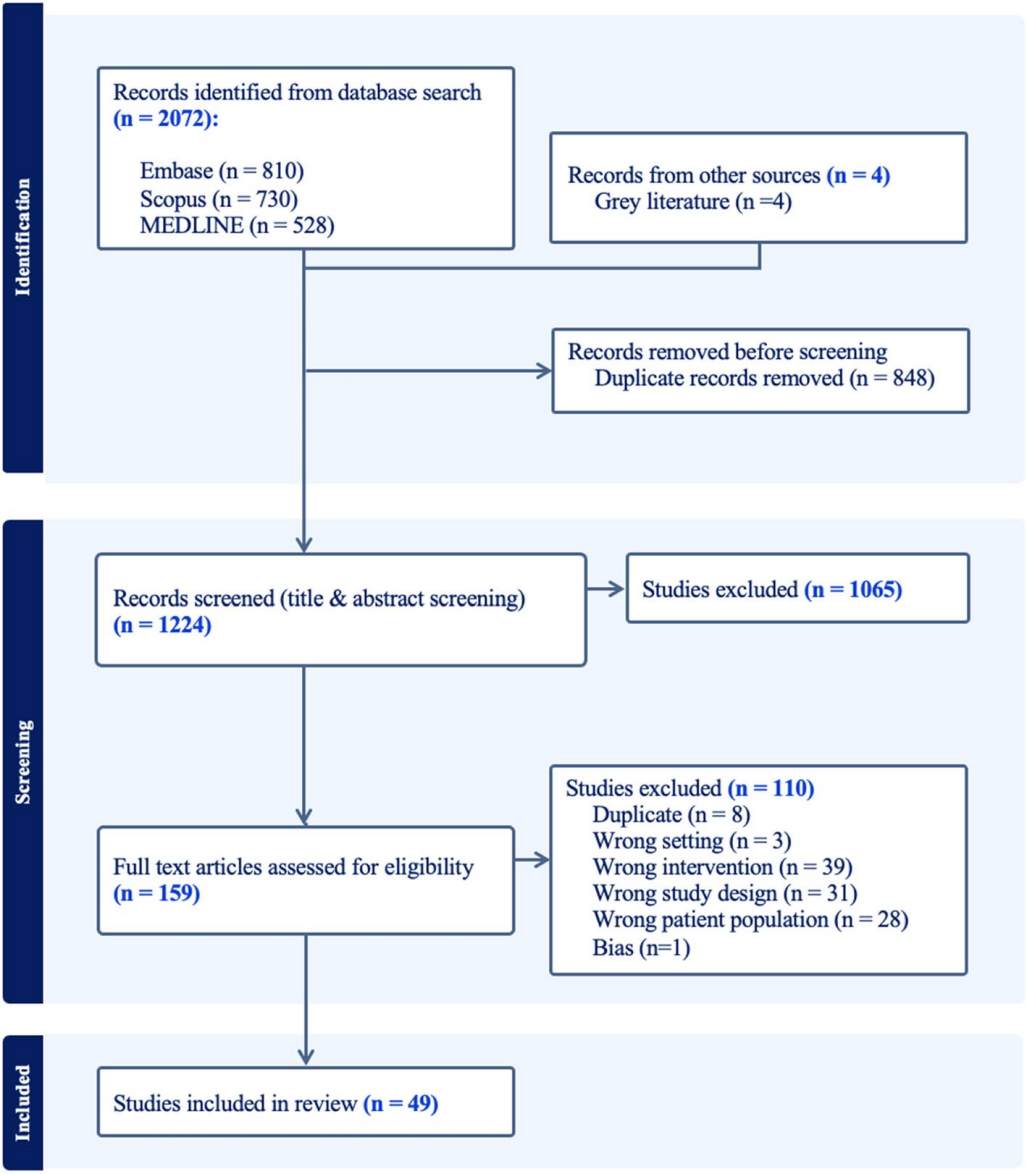
PRISMA diagram of study identification and selection

Our search yielded 2072 references, of which 848 duplicates were removed. Four additional literature sources were located and imported into COVIDENCE as grey literature. After title and abstract screening, 159 articles were retained. The full-text review of these articles resulted in 50 studies included from COVIDENCE. One qualitative study was excluded based on its risk of bias score, resulting in a total of 49 studies included. All studies recruited their participants through non-random sampling (including snowball, convenience, and purposive), resulting in a moderate risk of bias. A detailed risk of bias assessment for each study is presented in Additional file 1: Figure S1. The PRISMA diagram of study identification and selection is shown in Fig. 1.

### Characteristics of the included studies

**Fig. 2:**
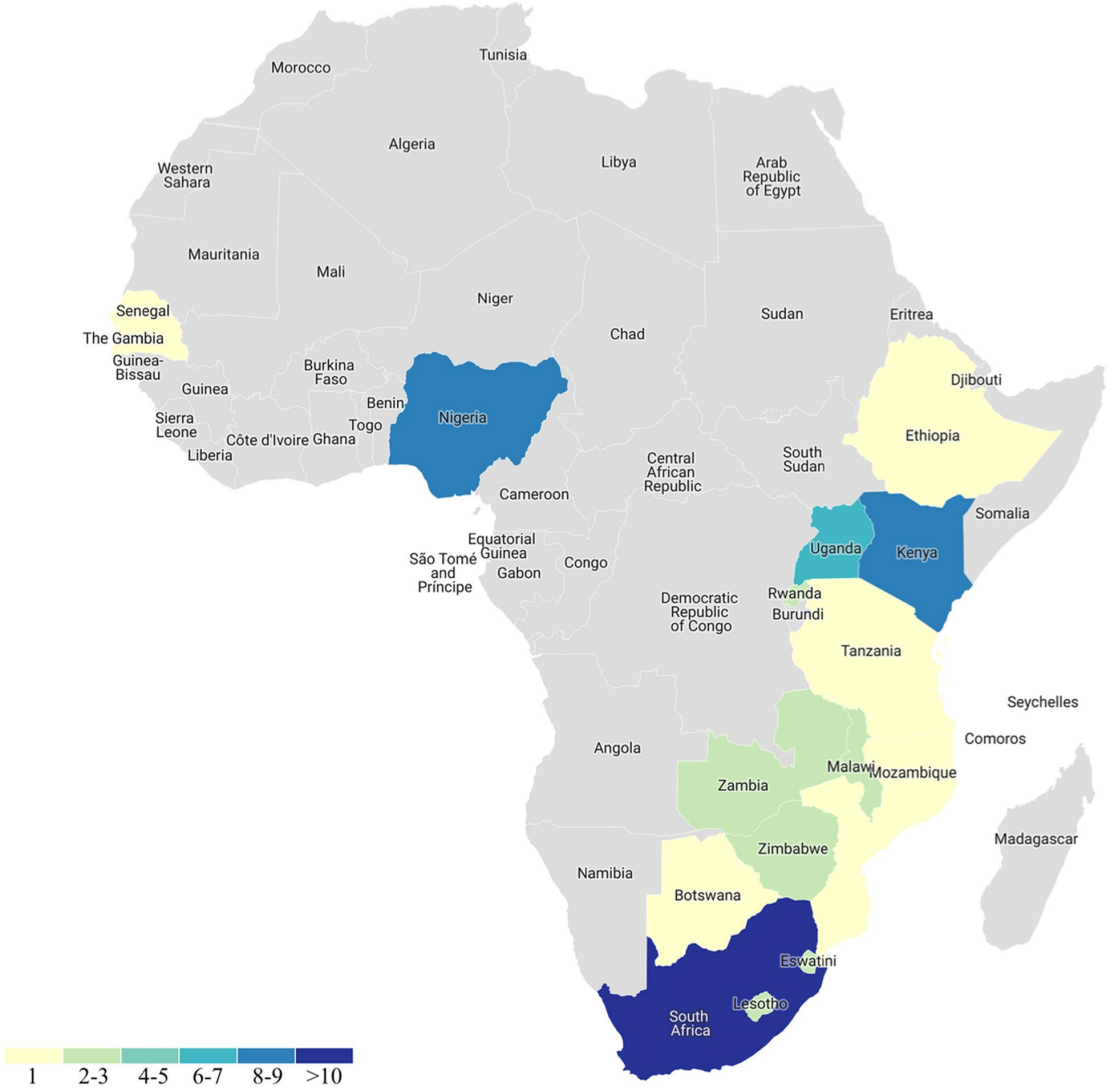
Geographic distribution of included studies

The characteristics of the 49 studies included in this review are summarized in Additional file 1: Table S3; 22 were cross-sectional, 10 were cohort, 10 were qualitative, four were from grey literature sources, and three were scoping reviews. The oldest study dates back to 2016, with the most recent published in 2023. All studies were conducted exclusively in Africa (Fig. 2): 31 in Southern Africa (South Africa, Mozambique, Zimbabwe, Malawi, Eswatini (Swaziland), Lesotho, Botswana, and Zambia), 19 in East Africa (Uganda, Kenya, Tanzania, and Rwanda), nine in West Africa (Nigeria and Senegal), and one in the horn of Africa (Ethiopia).

No studies conducted outside of sub-Saharan Africa met the review’s inclusion criteria. Notable absences existed in Northern and Central African studies, indicating their exclusion from this review. South Africa accounted for the highest representation with 17 studies, followed by Kenya, Nigeria, and Uganda with nine, eight, and seven studies included, respectively. While most studies were conducted within a single African country, four were multinational and took place in more than three African countries.

A synthesis of the included studies revealed two main themes and six subthemes. The main themes consist of (1) barriers to healthcare services, with the following subthemes: (i) availability, accessibility, and consistency-related barriers; (ii) individual, interpersonal, and structural barriers; and (iii) demographic and financial barriers; and (2) utilization patterns, with the following subthemes: (i) healthcare preferences; (ii) alternative healthcare modalities; and (iii) GAC.

### Barriers to healthcare services

#### Availability, accessibility, and consistency-related barriers

Within this review, 17 studies discussed the lack of available health services and resources tailored to TGD people [2, 26–41]. Participants highlighted that the insufficient availability of services tailored to TGD health challenged their access to appropriate care [27, 28]. According to a study by Mavhandu-Mudzusi et al. [29], participants reported that healthcare facilities failed to offer treatment for medical conditions frequently experienced by TGD people, such as anal thrush. GAC was also predominantly unavailable for many TGD people, as revealed in a South African study where only three public health facilities in the country offered such care [30].

Thirteen studies discussed the lack of recognition of TGD people in healthcare settings and the impact of cis-heterocentric services and beliefs on access to care [14, 27, 29–41]. Mavhandu-Mudzusi et al. [29] and Minor Peters [35] highlighted the connection between cis-heterocentric services, misgendering, and the imposition of binary gender expectations in healthcare settings and the direct obstruction of TGD people from accessing information about their unique healthcare needs and appropriate care. These studies revealed that some HCPs lacked an understanding of TGD identities, often conflating gender identity with sexual orientation. As a result, TGD women would be grouped with men who have sex with men and TGD men with women who have sex with women, leading to false assumptions about healthcare needs [27, 33]. Wolf [37] indicated that TGD people are frequently subsumed under a broad ‘homosexuality’ category, forcing TGD people to use services or participate in programs designed for men who have sex with men. The exclusion of TGD people in health care leads many to avoid seeking medical attention, even when their health is in critical condition [37].

Sixteen studies discussed the lack of HCPs possessing adequate knowledge of TGD healthcare needs [2, 14, 32, 35–38, 42–50]. This inadequacy was emphasized as a significant barrier to accessing care. Participants conveyed that HCPs often lacked the knowledge needed to provide optimum TGD care. Instances were reported where patients were turned away by providers unfamiliar with conditions commonly experienced by TGD people, such as anal thrush and anal sexually transmitted infections (STI) [2, 36]. In a study by van der Merwe et al. [32], 10% of participants felt that they almost always had to educate HCPs on TGD issues. Moreover, several South African studies have emphasized the scarcity of medical specialists and surgeons with adequate training to provide GAC, significantly impeding access to care for TGD people [35, 36].

Participants in seven studies discussed the limited accessibility of healthcare services [2, 27, 30, 37–39, 46]. In a study by Ssekamatte et al. [27], TGD sex workers reported prolonged wait times and limited operating hours at healthcare facilities. Additionally, the distance to healthcare centers emerged as a barrier to healthcare access. Poteat et al. [38] found that long travel times reduced participant interest in pre-exposure prophylaxis (PrEP). Interviews with TGD women revealed the frequent occurrence of insufficient supplies of TGD-specific medications, services, and necessary supplies, resulting in some participants being turned away without receiving any care [2, 27, 39]. Furthermore, two South African studies emphasized the lack of access to GAC, highlighting limitations such as medication shortages and waiting lists for gender-affirming surgery extending up to 25 years [35, 36].

Nine studies discussed barriers to the uptake and adherence of healthcare services [28, 39, 41–43, 50–53]. Respondents frequently reported two significant barriers to PrEP uptake and adherence: daily dosing patterns and human immunodeficiency virus (HIV)-related stigma [38, 41, 52]. Participants in five studies reported that daily medication intake, such as PrEP and antiretroviral therapy (ART), was inconvenient and led to skipping doses [38, 41, 42, 49, 53]. However, this barrier is not unique to TGD people. Another common barrier to adherence was medical mistrust due to inconsistent ART prescriptions and adherence messaging, as well as varying instructions regarding hormone dose adjustments while taking PrEP—leading to mistrust in HCPs and subsequent adverse adherence behaviours [38]. No studies were identified in regards to accessing oral healthcare services and patterns of dental service utilization by TGD people in Africa.

#### Individual, interpersonal, and structural barriers

Four studies discussed individualized stigma as a barrier to healthcare access and utilization [28, 43, 54, 55]. TGD women highlighted emotions of shame and low self-esteem that influenced their health-seeking behaviours, with some participants expressing concern about being unwelcomed by HCPs [28, 54]. Moreover, findings indicated that individualized stigma hinders access and utilization of gender-based violence support services by adversely impacting an individual’s self-perception and behaviours, such that they may become hesitant to seek care [50].

Thirty-six of the studies included in this research discussed healthcare-related stigma as a barrier, in addition to individualized stigma [2, 4, 26–32, 34, 36, 37, 39, 43, 44, 46–67]. HCP attitudes were frequently reported as a key barrier to healthcare access and utilization by TGD people, with some participants reporting that they did not receive the same level of care as cisgender patients [28]. Instances were described where providers refused care to TGD people,  prolonged wait times, denied medication access, and breached patient confidentiality [2, 27, 54]. Kimani et al. [50] noted that participants reported incidents of violence and invasive questioning by HCPs, which occurred in both public and private sectors. Participants from other studies reiterated this experience, reporting discrimination and shaming as drivers of their anxiety and fears when accessing healthcare services [54, 55]. Additionally, Wolf [37] highlighted instances where HCPs offered TGD people advice based on Christian religious beliefs rather than medical research due to the adverse ideology that TGD identity is an illness attributed to “demonic possession.” A study by Mbeda et al. [61] identified the most frequently reported consequence of healthcare-related stigma as the fear of seeking healthcare services. This finding was consistent across other studies; participants reported delaying or avoiding seeking care and fear of disclosing their identity or hiding their identity from HCPs. This fear stemmed from concerns about negative experiences, consequently restricting access to appropriate prevention and care services [26, 34].

Criminalization and oppressive legislature were discussed in five studies [29, 48, 68–70]. Findings revealed that criminalization directly and indirectly affects healthcare service access. Some TGD people may delay seeking care and treatment, while others may face barriers like community discrimination that prevent them from reaching health facilities [68]. Several nurses in a study by Muwanguzi et al. [48] expressed reluctance, fearing criminal complicity, and mentioned their inclination to involve the police when encountering TGD people.

#### Demographic and financial barriers

In seven of the included studies, participants indicated that financial constraints deterred them from accessing desired services, such as PrEP and GAC [27, 35, 38, 44, 46, 47, 50]. The inability to afford medications, treatment, medical bills, and transportation to and from healthcare facilities limited participants’ access to care [27]. Two studies highlighted how the lack of insurance coverage for various healthcare services, including GAC, prevented service access [38, 47]. One study reported that the most common reason individuals chose not to take PrEP was a lack of medical insurance coverage [38]. The level of urbanization was also discussed as a barrier to healthcare access in four studies. Participants emphasized that although GAC was available in both the public and private sectors, this access was limited to urban centers [36, 56].

### Patterns of healthcare utilization

#### Healthcare preferences

Six studies discussed healthcare preferences among TGD people [2, 14, 33, 41, 52, 55]. Most studies indicated a preference for private over public facilities, believing that HCPs in private facilities respected patient privacy and provided higher-quality care [2, 14, 41, 66]. Another preference, found in one quantitative study, revealed that many TGD participants preferred the use of new longer-acting formulations of PrEP—particularly subdermal implants—over other PrEP options due to the longer duration of protection, longer periods between clinic visits, the perceived safety in conjunction with hormone therapy, and the more ‘feminine’ feeling it provided [53].

#### Alternative healthcare modalities

Patterns of alternative healthcare service utilization were discussed in seven studies, with self-medication specifically addressed in all seven of them [2, 14, 33, 41, 42, 46, 51]. An alarming 79% of participants in a study by Kombol [46] admitted to engaging in self-medication. Findings from this review suggested that self-medication often arose due to limited access to healthcare services and medications or due to the fact that HCPs were either unwilling or lacked the competence to deliver necessary care [2, 14, 33]. Furthermore, findings from another review noted challenges encountered by TGD people within conventional healthcare settings, which prompted them to turn to alternative sources, such as traditional healers, peers, or other untrained personnel, to manage their healthcare needs [46].

#### Gender-affirming care

The inaccessibility of GAC, including GHAT and surgeries, was discussed in six studies [4, 27, 30, 32, 36, 45]. Evidence from this review indicated low use of GAC. Cumulative data from nine countries revealed that only 31% of participants had access to GAHT, and only 22% had access to surgical procedures [30] Nearly half the participants (49%) in a study by Van der Merwe et al. [32] expressed a desire to affirm their gender with hormones; however, a minority (11%) had accessed such hormones. Mujugira et al. [4] found that only a small minority of TGD participants (18%) had ever used hormone therapy and Ssekamatte et al. [27] indicated that access to GAC was limited due to frequent medication shortages [27]. Limited time for operating room procedures also resulted in waiting lists for gender-affirming surgeries that extend beyond two decades [36]. Only one percent of participants in a study by Van der Merwe et al. [32] had undergone gender-affirming breast enhancement, despite 62% expressing a desire for it.

GAHT and surgeries are primarily accessed through the private sector where available [27, 36]. However, findings indicated that the black market, online sources, and private pharmacies were also used to obtain GAHT [4]. Furthermore, the results of this review indicated that the use of GAHT may influence health-related decision-making. Some TGD patients expressed concerns about the coadministration of hormones with PrEP and ART use due to perceptions of potential interaction [37, 53].

## Discussion

Our study aimed to consolidate existing evidence on the access to healthcare services and utilization of GAC among TGD people in Africa. Understanding the intersectional factors that act as barriers to equitable healthcare access for TGD people is essential for developing comprehensive and inclusive healthcare interventions and policies. This systematic review revealed numerous barriers related to service availability, accessibility, and consistency, as well as individualized, interpersonal, structural, demographic, and financial factors that impede healthcare access and utilization. This discussion will provide an overview of our findings regarding potential negative consequences resulting from inadequate access to healthcare services and GAC.

Our review revealed limited evidence regarding the barrier to care and the high treatment needs of TGD people in Africa. There was a significant disparity in the distribution of healthcare services across different regions of the continent, with most of our included studies focusing on South Africa, Kenya, Nigeria, and Uganda. This highlights a noticeable lack of published research in Northern and Central African countries. This unequal distribution could be due to various geopolitical factors, such as the existence of strict laws regarding TGD people and other minority genders, as well as diverse attitudes towards TGD people across different African regions. Furthermore, our findings reveal a lack of emphasis on research initiatives aimed at improving TGD health outcomes and inadequate access to healthcare services and GAC for TGD people in Africa.

Our review found that the study design of the identified studies lacked robustness, particularly regarding participant recruitment and data collection. All these studies used non-random sampling methods, such as snowball, convenience, and purposive sampling, to recruit participants. Only one study claimed to have achieved generalizable results. This difference in achieving generalizability can be attributed to the limited accessibility of TGD people for research purposes [32, 71]. The TGD population in various African countries often encounters high levels of stigma and discrimination, making many individuals hesitant or fearful about participating in research studies [32]. This hesitation may stem from the potential repercussions of disclosing their gender identity, raising concerns about the reliability of findings drawn from non-random samples [26, 34, 72]. Relying solely on these samples to represent the entire TGD population may introduce significant bias, and fail to accurately capture the diverse experiences, needs, and barriers faced by this key population. Selecting biased samples can also skew results and perpetuate misconceptions about healthcare access and utilization among TGD people, further highlighting the importance of using representative samples in research [1]. However, it is essential to note that among these reliability risks, non-random sampling may be the most suitable option for the collection of data among TGD people in the current context as it will aid in the obtainment of a larger sampling size, facilitating the analysis of TGD people with different gender identities [73].

Of the 49 studies included, the majority revealed barriers related to the availability, accessibility, and consistency of healthcare services [27–29]. Furthermore, the issue of healthcare-related stigma was found to be pervasive among this population, with participants reporting experiences such as encountering uninformed HCPs, a lack of tailored resources for TGD health [27, 28], and distressing encounters including refusal of care, prolonged wait times, denial of medication access, and breaches of patient confidentiality. The repercussions of these discriminatory encounters extended far beyond mere inconveniences—they amplified emotions of shame, anxiety, and fears among TGD people when seeking healthcare services [26, 34, 54, 56]. Continued mistreatment of TGD people significantly impacts their trust in the healthcare system [38]. Evidence suggests that this exclusionary approach often leads to hesitation among TGD people in seeking timely medical care, even when faced with critical health conditions [37]. This avoidance or delay in seeking appropriate care exacerbates existing health disparities, prolongs suffering, and can lead to the development of preventable or treatable health conditions [74]. The exclusionary practices and cis-heterocentric environment not only perpetuate this cycle but also silence and marginalize the voices and representation of TGD people, particularly in countries like Mauritania, Nigeria, Uganda, and South Sudan, where LGBTQ + discrimination is reinforced by colonial-era laws that have been instilled for half a century [31, 33].

Financial and demographic barriers were also identified in Kenya, South Africa, and Uganda. These barriers specifically pertain to an individual’s ability to access healthcare services and medication adherence due to financial constraints. The inability to afford crucial medications, medical bills, transportation, and insurance coverage prevents many TGD people from receiving necessary healthcare [35, 38]. As a result, TGD people may skip doses or forego important medical procedures, negatively impacting their health outcomes and exacerbating existing ones. These financial challenges are more prevalent in rural rather than urban regions, hindering access to GAC availability in urban centers.

Our review found that TGD people in Uganda and South Africa preferred private healthcare facilities over public ones [33, 36]. This inclination may be attributed to the perceived efficiency and higher quality of private facilities and their profit-driven nature. Many participants believed that the profit motive would incentivize HCPs to overlook their gender identity when treating them [33]. However, despite this preference, TGD people consistently reported inadequate care within these settings, particularly when seeking reproductive and sexual health services [33]. The root of this inadequacy lies in the fear of disclosing health-related concerns due to HCPs’ lack of sensitivity and knowledge about the unique needs of TGD people. Moreover, the concern about sharing health concerns was exacerbated by HCPs inquiring about the gender identity of TGD people. This can create discomfort, especially in settings where discrimination is commonly reported in literature. Thus, accessing adequate healthcare may be challenging for these individuals, even at private facilities. This cannot be generalized, however, as evidence from North and Central Africa is missing.

Many studies identified patterns of alternative healthcare service utilization, including self-medication. An alarming trend of people engaging in self-medication has been identified, linking itself to several factors, such as limited access to healthcare services and medication, reluctance, or lack of knowledge among HCPs to provide necessary care, and fear of discrimination or stigmatization when seeking care [46, 54, 56]. As a result, many TGD people turn to unconventional sources to address their healthcare needs despite the potential risks and harm associated with unregulated practices [46].

Our review identified the inaccessibility of GAC, including hormone replacement therapy and gender-affirming surgery, as an important topic [27, 30, 32, 36]. Despite most participants expressing a desire for GHAT to affirm their gender identity, only a small minority had access to it [32] An even smaller minority underwent gender-affirming surgery despite a majority expressing a desire for it [32]. The primary contributing factor to this limited utilization is the lack of accessibility. While these treatments are primarily available through private healthcare facilities, financial constraints, fear of discrimination and harsh laws act as barriers preventing many TGD people from accessing them. Furthermore, the exclusion of GAC from health insurance coverage exemplifies how TGD healthcare needs are deemed unimportant [36]. The lack of accessible, affordable, and affirming care can lead individuals toward unsupervised self-medication and illicit hormone use [2, 14]. Failing to integrate GAC within health centers not only places TGD patients at risk for preventable health complications but also prompts them to resort to alternative, potentially hazardous, means to affirm their gender and manage their healthcare needs.

Results from this review highlight the need for advocacy and further research to address the unmet healthcare needs of TGD in Africa. The results further highlight the need for sensitivity training among HCPs to promote inclusive services and reduce stigma and discrimination. Additionally, the implementation of specialized curricula for HCPs focused on LGBTQ + individuals is necessary to address the reported lack of knowledge regarding trans-specific healthcare needs.

This systematic review has limitations. In several instances, the studies combined men who have sex with men and TGD women groups, presenting their findings jointly despite acknowledging the distinctions between these two groups. Moreover, most of the studies utilized non-random sampling, which, while beneficial for engaging high-risk populations, may limit the generalizability of their results. Additionally, limited evidence was available from various African regions, particularly North and Central Africa. Laws, healthcare provisions, and attitudes towards TGD people also vary across different African regions; therefore, results from one country may not reflect others.

## Conclusions

This systematic review is the first attempt to consolidate existing evidence regarding comprehensive and inclusive healthcare services among TGD people in Africa. The review identified a spectrum of barriers that impede healthcare access and utilization within TGD communities across the continent. These barriers include limited service availability, lack of accessibility and consistency, and demographic, financial, individual, interpersonal, and structural factors. This intersection of barriers significantly contributes to the disproportionate burden of disease faced by TGD people. The available literature illustrates the far-reaching consequences of these barriers on healthcare utilization and service preferences among TGD people in Africa and the need to prioritize programs aimed at reducing such barriers.

### Supplementary Information

Additional file 1: Table S1: PRISMA checklist. Table S2: Search strategy. Figure S1. Risk of bias assessment. Table S3: Summary of studies meeting the inclusion criteria.

## Data Availability

This systematic review is based on published literature. All data generated are available in the main text or supplementary material.

## Abbreviations

AACODS: Authority, Accuracy, Coverage, Objectivity, Date and Significance
ART: Antiretroviral therapy
GAC: Gender-affirming care
GAHT: Gender-affirming hormone therapy
HCP: Healthcare provider
HIV: Human immunodeficiency virus
JBI: Joanna Briggs Institute
LGBTQ +: Lesbian, gay, bisexual, transgender, queer, and other sexual minorities
PrEP: Pre-exposure prophylaxis
PRISMA: Preferred Reporting Items for Systematic Reviews and Meta-Analyses
STI: Sexually transmitted infections
TGD: Transgender and gender diverse
