# Supplementary material for: Healthcare access and barriers to utilization among transgender and gender diverse people in Africa: a systematic review
Source: BMC Glob Public Health. 2024 Jun 27;2:44. doi: 10.1186/s44263-024-00073-2 (PMC11208260; doi:10.1186/s44263-024-00073-2)
Supplement: Supplementary file 1 — Additional file 1: Table S1: PRISMA checklist. Table S2: Search strategy. Figure S1. Risk of bias assessment. Table S3: Summary of studies meeting the inclusion criteria. [file 44263_2024_73_MOESM1_ESM.docx]

**Additional file 1: Table S1.** PRISMA checklist

| **Section and Topic** | **Item #** | **Checklist item** | **Location where item is reported** |
| --- | --- | --- | --- |
| **TITLE** | | |  |
| Title | 1 | Identify the report as a systematic review. | Page 1 |
| **ABSTRACT** | | |  |
| Abstract | 2 | See the PRISMA 2020 for Abstracts checklist. | Pages 2-3 |
| **INTRODUCTION** | | |  |
| Rationale | 3 | Describe the rationale for the review in the context of existing knowledge. | Pages 5-7 |
| Objectives | 4 | Provide an explicit statement of the objective(s) or question(s) the review addresses. | Pages 7-8 |
| **METHODS** | | |  |
| Eligibility criteria | 5 | Specify the inclusion and exclusion criteria for the review and how studies were grouped for the syntheses. | Page 9 |
| Information sources | 6 | Specify all databases, registers, websites, organisations, reference lists and other sources searched or consulted to identify studies. Specify the date when each source was last searched or consulted. | Page 8 |
| Search strategy | 7 | Present the full search strategies for all databases, registers and websites, including any filters and limits used. | Additional file 2: Table S2 |
| Selection process | 8 | Specify the methods used to decide whether a study met the inclusion criteria of the review, including how many reviewers screened each record and each report retrieved, whether they worked independently, and if applicable, details of automation tools used in the process. | Pages 8-9 |
| Data collection process | 9 | Specify the methods used to collect data from reports, including how many reviewers collected data from each report, whether they worked independently, any processes for obtaining or confirming data from study investigators, and if applicable, details of automation tools used in the process. | Pages 9-10 |
| Data items | 10a | List and define all outcomes for which data were sought. Specify whether all results that were compatible with each outcome domain in each study were sought (e.g. for all measures, time points, analyses), and if not, the methods used to decide which results to collect. | Page 10 |
|  | 10b | List and define all other variables for which data were sought (e.g. participant and intervention characteristics, funding sources). Describe any assumptions made about any missing or unclear information. | Pages 9-10 |
| Study risk of bias assessment | 11 | Specify the methods used to assess risk of bias in the included studies, including details of the tool(s) used, how many reviewers assessed each study and whether they worked independently, and if applicable, details of automation tools used in the process. | Pages 9-10 |
| Effect measures | 12 | Specify for each outcome the effect measure(s) (e.g. risk ratio, mean difference) used in the synthesis or presentation of results. | N/A |
| Synthesis methods | 13a | Describe the processes used to decide which studies were eligible for each synthesis (e.g. tabulating the study intervention characteristics and comparing against the planned groups for each synthesis (item #5)). | Page 10 |
|  | 13b | Describe any methods required to prepare the data for presentation or synthesis, such as handling of missing summary statistics, or data conversions. | Page 10 |
|  | 13c | Describe any methods used to tabulate or visually display results of individual studies and syntheses. | Page 10 |
|  | 13d | Describe any methods used to synthesize results and provide a rationale for the choice(s). If meta-analysis was performed, describe the model(s), method(s) to identify the presence and extent of statistical heterogeneity, and software package(s) used. | Pages 10 |
|  | 13e | Describe any methods used to explore possible causes of heterogeneity among study results (e.g. subgroup analysis, meta-regression). | N/A |
|  | 13f | Describe any sensitivity analyses conducted to assess robustness of the synthesized results. | N/A |
| Reporting bias assessment | 14 | Describe any methods used to assess risk of bias due to missing results in a synthesis (arising from reporting biases). | Pages 10-11 |
| Certainty assessment | 15 | Describe any methods used to assess certainty (or confidence) in the body of evidence for an outcome. | N/A |
| **RESULTS** | | |  |
| Study selection | 16a | Describe the results of the search and selection process, from the number of records identified in the search to the number of studies included in the review, ideally using a flow diagram. | Figure 1 |
|  | 16b | Cite studies that might appear to meet the inclusion criteria, but which were excluded, and explain why they were excluded. | Figure 1 |
| Study characteristics | 17 | Cite each included study and present its characteristics. | Additional file 4: Table S3 |
| Risk of bias in studies | 18 | Present assessments of risk of bias for each included study. | Additional file 3: Figure S1 |
| Results of individual studies | 19 | For all outcomes, present, for each study: (a) summary statistics for each group (where appropriate) and (b) an effect estimate and its precision (e.g. confidence/credible interval), ideally using structured tables or plots. | Additional file 4: Table S3 |
| Results of syntheses | 20a | For each synthesis, briefly summarise the characteristics and risk of bias among contributing studies. | Additional file 3: Figure S1 |
|  | 20b | Present results of all statistical syntheses conducted. If meta-analysis was done, present for each the summary estimate and its precision (e.g. confidence/credible interval) and measures of statistical heterogeneity. If comparing groups, describe the direction of the effect. | N/A |
|  | 20c | Present results of all investigations of possible causes of heterogeneity among study results. | N/A |
|  | 20d | Present results of all sensitivity analyses conducted to assess the robustness of the synthesized results. | N/A |
| Reporting biases | 21 | Present assessments of risk of bias due to missing results (arising from reporting biases) for each synthesis assessed. | N/A |
| Certainty of evidence | 22 | Present assessments of certainty (or confidence) in the body of evidence for each outcome assessed. | N/A |
| **DISCUSSION** | | |  |
| Discussion | 23a | Provide a general interpretation of the results in the context of other evidence. | Pages 17-21 |
|  | 23b | Discuss any limitations of the evidence included in the review. | Pages 21-22 |
|  | 23c | Discuss any limitations of the review processes used. | Pages 21-22 |
|  | 23d | Discuss implications of the results for practice, policy, and future research. | Page 22 |
| **OTHER INFORMATION** | | |  |
| Registration and protocol | 24a | Provide registration information for the review, including register name and registration number, or state that the review was not registered. | Page 8 |
|  | 24b | Indicate where the review protocol can be accessed, or state that a protocol was not prepared. | Page 8 |
|  | 24c | Describe and explain any amendments to information provided at registration or in the protocol. | N/A |
| Support | 25 | Describe sources of financial or non-financial support for the review, and the role of the funders or sponsors in the review. | Page 23 |
| Competing interests | 26 | Declare any competing interests of review authors. | Page 23 |
| Availability of data, code and other materials | 27 | Report which of the following are publicly available and where they can be found: template data collection forms; data extracted from included studies; data used for all analyses; analytic code; any other materials used in the review. | Page 23 |

*From:*  Page MJ, McKenzie JE, Bossuyt PM, Boutron I, Hoffmann TC, Mulrow CD, et al. The Prisma 2020 statement: An updated guideline for reporting systematic reviews [Internet]. British Medical Journal Publishing Group; 2021 [cited 2024 Jun 10]. Available from: https://www.bmj.com/content/372/bmj.n71

**Additional file 1: Table S2.** Search strategy

|  | Database | | |
| --- | --- | --- | --- |
| Concept | PubMed/MEDLINE | Scopus | Embase |
| Transgender | exp transgender/ or (person, transgender or person, transgendered or person, transsexual or person, two-spirit or persons, transgendered or transexual or transexuals or transgender or transgender person or transgender persons or transgendered person or transgendered persons or transgenders or transsexual person or transsexual persons or two spirit persons or two-spirit person or gender non conforming or "gender minorit*").ab,ti. | ( ( ABS ( ( "person, transgender" OR "person, transgendered" OR "person, transsexual" OR "person, two-spirit" OR "persons, transgendered" OR transexual OR transexuals OR transgender OR "transgender person" OR "transgender persons" OR "transgendered person" OR "transgendered persons" OR transgender* OR "transsexual person" OR "transsexual persons" OR "two spirit persons" OR "two-spirit person" OR "gender non conforming" OR "gender minorit*" ) ) OR TITLE ( ( "person, transgender" OR "person, transgendered" OR "person, transsexual" OR "person, two-spirit" OR "persons, transgendered" OR transexual OR transexuals OR transgender OR "transgender person" OR "transgender persons" OR "transgendered person" OR "transgendered persons" OR transgender* OR "transsexual person" OR "transsexual persons" OR "two spirit persons" OR "two-spirit person" OR "gender non conforming" OR "gender minorit*" ) ) ) ) ) | exp transgender/ or (person, transgender or person, transgendered or person, transsexual or person, two-spirit or persons, transgendered or transexual or transexuals or transgender or transgender person or transgender persons or transgendered person or transgendered persons or transgenders or transsexual person or transsexual persons or two spirit persons or two-spirit person or gender non conforming or "gender minorit*").ab,ti. |
| Access | (Access* or barrier* or challeng* or facilitat* or health* or utili* or dent* or oral or service* or sex* or reproduct* or fertil* or counsel* or mental* or sociodemographic*).ab,ti. | ( ( ABS ( ( access* OR barrier* OR challeng* OR facilitat* OR sociodemographic* OR stigma* OR discriminat* ) ) OR TITLE ( ( access* OR barrier* OR challeng* OR facilitat* OR sociodemographic* OR stigma* OR discriminat* ) ) ) ) | (Access* or barrier* or challeng* or facilitat* or health* or utili* or dent* or oral or service* or sex* or reproduct* or fertil* or counsel* or mental* or sociodemographic*).ab,ti. |
| Africa | exp Africa/ or (africa* or sub-sahara* or "global south").ab,ti. | ( ( ABS ( ( africa* OR “sub-sahara*” OR “global south” ) ) OR TITLE ( ( africa* OR “sub- sahara*” OR “global south” ) ) | exp Africa/ or (africa* or sub-sahara* or "global south").ab,ti. |
|  | 1 AND 2 AND 3  n = 528 | 1 AND 2 AND 3  n = 730 | 1 AND 2 AND 3  n = 810 |

**Additional file 1: Figure S1.** Risk of bias assessment for each included study using the CLARITY cross sectional tool, CLARITY cohort tool, JBI qualitative study tool, JBI systematic review tool, and AACODS checklist


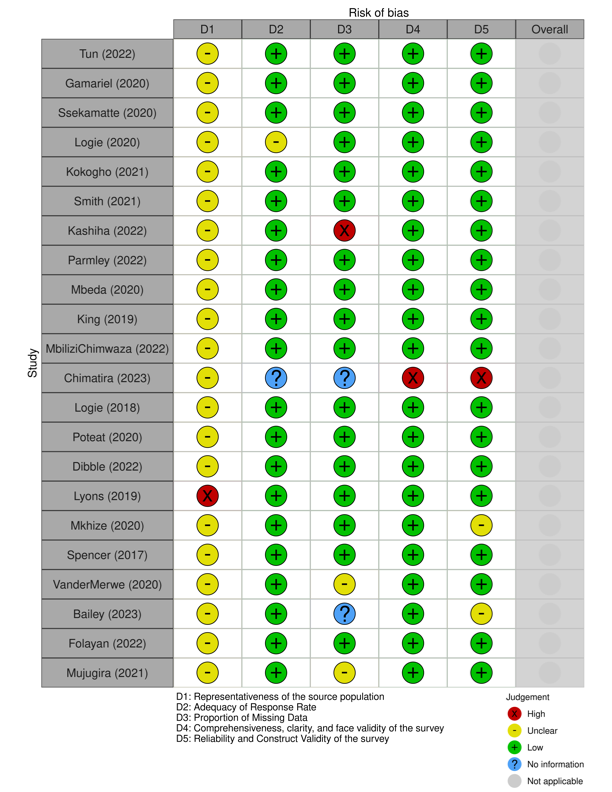


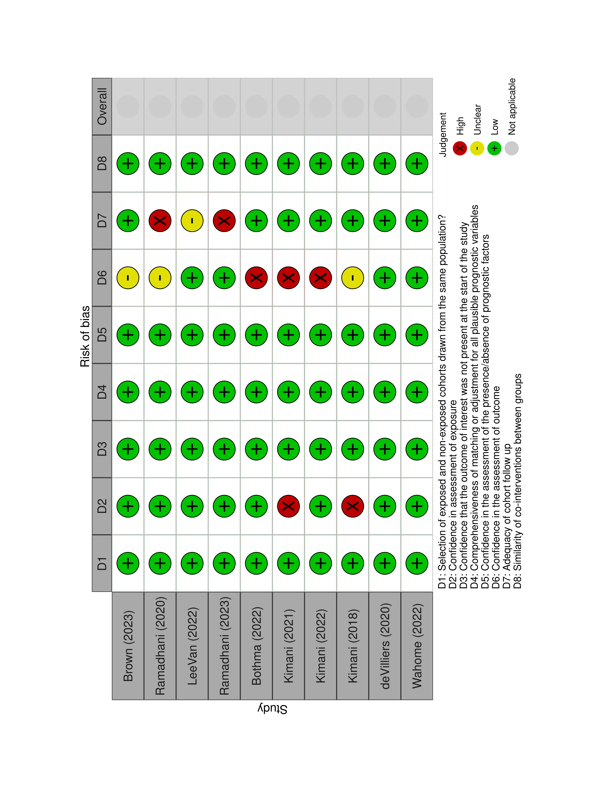


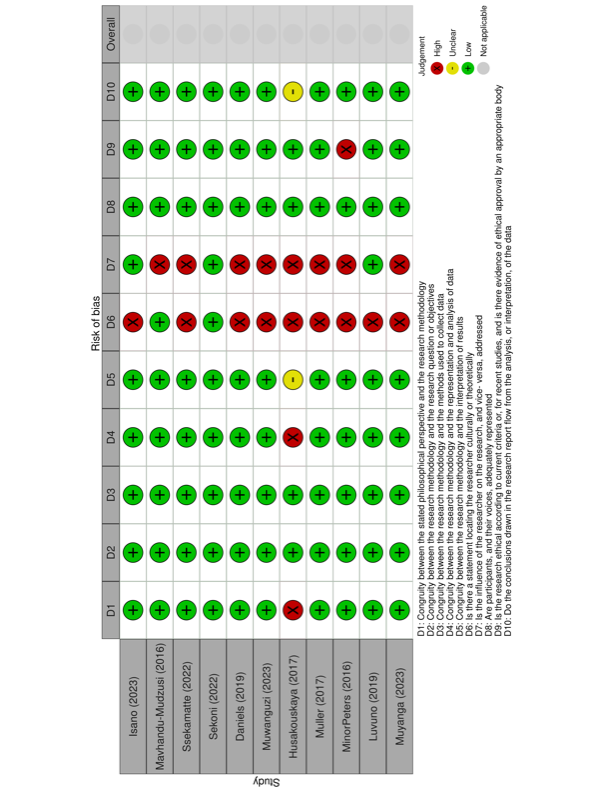


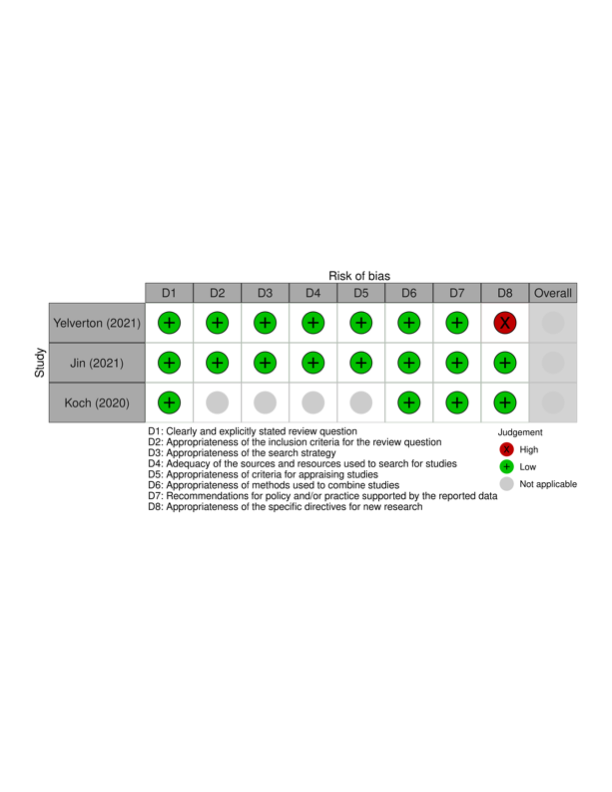


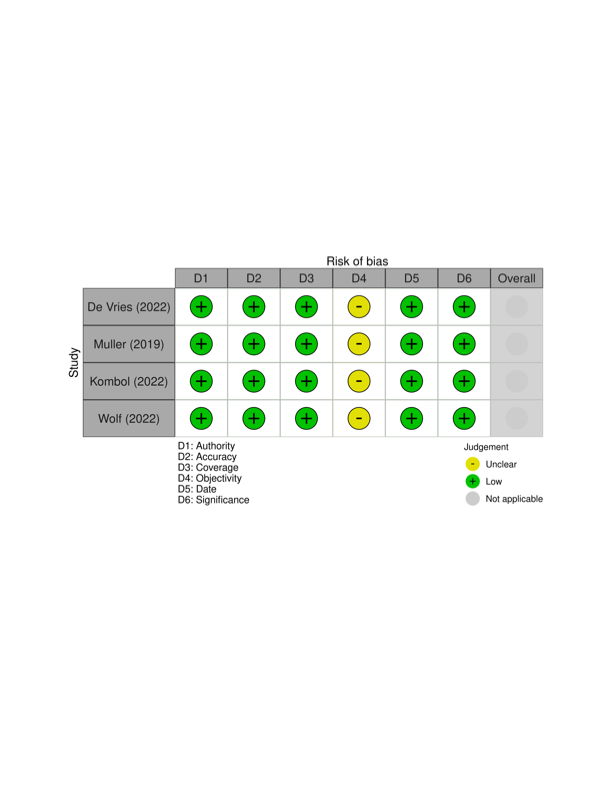


**Additional file 1: Table S3.** Summary of studies meeting the inclusion criteria

| **Study** | **Location** | **Study design** | **Data collection tool** | **Sampling method** | **Total sample size** | **Theme of the manuscript** |
| --- | --- | --- | --- | --- | --- | --- |
| Bailey et al., 2023 | Kenya | Cross-sectional | Interview | Convenience & purposive sampling | 423 | HIV prevention |
| Bothma et al., 2022 | South Africa | Cohort | Self-reported questionnaire | Convenience & purposive sampling | 5,636 | Differentiated HIV services |
| Brown et al., 2023 | South Africa | Prospective cohort study | Survey | Convenience sampling | 201 | Association of stigma and HIV service and prevention uptake |
| Chimatira et al., 2023 | South Africa | Cross-sectional | Survey | Simple random sampling | 249 | Human rights violations |
| Daniels et al., 2019 | South Africa | Cross-sectional | Interview | Convenience sampling | 20 | Relationship desire and medical mistrust |
| de Villiers et al., 2020 | South Africa | Cohort study | Interview | Purposively sampled | 10 | Stigma and HIV service access |
| de Vries & McLachlan, 2022 | South Africa | Scholarly article | / | / | / | Access to gender-affirming care |
| Dibble et al., 2022 | Senegal | Cross-sectional | Interview | Respondent-driven sampling | 724 | Stigma and healthcare access |
| Folayan et al., 2022 | Nigeria | Cross-sectional | Self-reported questionnaire | Snowball sampling | / | Rights violations |
| Gamariel et al., 2020 | Mozambique | Cross-sectional | Interview | Purposive & snowball sampling | 27 | Access to health services |
| Isano et al., 2023 | Rwanda | Phenomenological approach | Interview | Purposive & snowball sampling | 28 | Healthcare-seeking experiences |
| Jin et al., 2021 | Africa | Scoping review | / | / | / | Epidemiological conditions of HIV |
| Kashiha & Rider, 2022 | Tanzania | Cross-sectional | Self-reported questionnaire & interview | Snowball, convenience, & purposive sampling | 300 | Health access |
| Kimani et al., 2019 | Kenya | Cohort study | Interview | Convenience sample | 168 | PrEP interest and HIV-1 incidence |
| Kimani et al., 2021 | Kenya | Cohort | Interview | Purposive sampling | 53 | PrEP adherence and persistence |
| Kimani et al., 2022 | Kenya | Cohort study | Interview | Purposive sampling | 21 | HIV prevention |
| King et al., 2019 | Uganda | Cross-sectional | Interview | Snowball sampling | 45 | HIV and gender-related contexts of transgender women |
| Koch et al., 2020 | South Africa | Literature review | / | / | / | Cost of being transgender |
| Kokogho et al., 2021 | Nigeria | Cross-sectional | Interview | Respondent-driven sampling | 2,557 | Condom use and stigma |
| Kombol, 2022 | Nigeria | Scholarly article | / | / | / | Access to healthcare |
| LeeVan et al., 2022 | Nigeria | Prospective, Observational cohort | Interview | Respondent-driven sampling | 838 | Health burden |
| Logie et al., 2018 | Swaziland | Cross-sectional | Interview | Purposive, snowball, & convenience sampling | 51 | Marginalization and social change processes |
| Logie et al., 2020 | Lesotho | Cross-sectional | Interview | Purposive & Convenience sampling | 46 | Stigma and HIV vulnerabilities |
| Luvuno et al., 2019 | South Africa | Phenomenological and critical ethnographic approaches | Interview | Purposive & snowball sampling | 16 | Access to reproductive healthcare |
| Lyons et al., 2019 | Eswatini | Cross-sectional | Self-reported questionnaire | Chain-referral-sampling | 532 | Stigma and outness about sexual behaviours |
| Mavhandu-Mudzusi, 2016 | South Africa | Interpretative phenomenological analysis | Interview | Snowball sampling | 20 | Citizenship rights, discrimination, and stigmatization |
| Mbeda et al., 2020 | Kenya, Malawi, & South Africa | Cross-sectional | Interview | Snowball, convenience, & purposive sampling | 401 | Health-care related stigma |
| Mbilizi Chimwaza et al., 2022 | Kenya, Malawi, &  South Africa | Cross-sectional | Interview | Purposive sampling | 80 | HIV risk perception and sexual behaviour |
| Minor Peters, 2016 | Uganda | Ethnographic study | Interview | Snowball sampling | 50 | HIV prevention and treatment |
| Mkhize & Maharaj, 2020 | South Africa | Cross-sectional | Interview | Purposive & snowball sampling | 12 | Structural violence and access to health services |
| Mujugira et al., 2021 | Uganda | Cross-sectional | Interview | Snowball sampling | 50 | Transgender stigma and risk of STIs |
| Müller, 2017 | South Africa | Grounded theory | Interview | Snowball sample | 16 | Availability, acceptability, accessibility, and quality of healthcare |
| Muwanguz et al., 2023 | Uganda | Cross-sectional | Interview | Purposive sampling | / | Reflection on sexual and gender minorities |
| Muyanga et al., 2023 | Uganda | Narrative inquiry | Interview | Snowball & purposive sampling | 60 | Gender-based violence and utilization of HIV interventions |
| Parmley et al., 2022 | Zimbabwe | Cross-sectional | Self-reported questionnaire | Respondent-driven sampling | 718 | HIV infection prevalence |
| Poteat et al., 2020 | South Africa | Cross-sectional | Interview | Convenience sample | 213 | PrEP awareness and engagement |
| Ramadhani et al., 2020 | Nigeria | Observational cohort | Interview | Respondent-driven sampling | 2,123 | Age and healthcare needs and engagement |
| Ramadhani et al., 2023 | Nigeria | Cohort | Self-reported questionnaire | Convenience sampling | 788 | Determinants of preexposure prophylaxis |
| Sekoni et al., 2022 | Nigeria | Cross-sectional | Interview | Respondent-driven sampling | 35 | Experiences of LGBT+ people |
| Smith et al., 2021 | Kenya | Cross-sectional | Self-reported questionnaire | Respondent-driven sampling | 618 | Disparities and care coverage |
| Spencer et al., 2017 | South Africa | Cross-sectional | Interview | Snowball sampling | 12 | Gender-affirming care |
| Ssekamatte et al., 2020 | Uganda | Cross-sectional | Interview | Purposively & snowball sampling | 22 | Access and utilization of HIV/STIs services |
| Ssekamatte et al., 2022 | Uganda | Cross-sectional | Interview | Snowball sampling | 60 | Gender-based violence help-seeking and challenges |
| Tun et al., 2022 | Nigeria | Cross-sectional | Interview | Convenience & purposive sampling | 38 | Influence of stigma on HIV services |
| van der Merwe et al., 2020 | South Africa | Cross-sectional | Interview | Convenience & snowball sampling | 383 | Socioeconomic determinants of health for transgender women |
| Wahome et al., 2022 | Kenya | Prospective cohort study | Interview & self-reported questionnaire | Convenience sample | 134 | PrEP adherence |
| Wolf, 2022 | Rwanda | Scholarly article | / | / | / | Healthcare access |
| Yelverton et al., 2021 | Zambia | Literature review | / | / | / | HIV care access |
